# Orthostatic hypotension secondary to a suspected thymoma in a dog: a case report

**DOI:** 10.1186/s12917-020-02604-z

**Published:** 2020-10-13

**Authors:** Jeremy Hansford, Natalia Henao-Guerrero

**Affiliations:** Department of Small Animal Clinical Sciences, Virginia Maryland College of Veterinary Medicine, Blacksburg, VA 24061 USA

**Keywords:** Thymoma, Orthostatic hypotension, Positional hypotension, Phenylephrine

## Abstract

**Background:**

This is the first case report description, to our knowledge, of a cranial mediastinal mass (suspected thymoma) causing orthostatic hypotension in a dog.

**Case presentation:**

A Labrador Retriever presented for urethral stent placement during cystoscopy secondary to transitional cell carcinoma diagnosis. During anesthesia, the patient had unexpected severe and poorly-responsive hypotension following a shift in position. Several days later, an intrathoracic mass was discovered, raising concerns that the position of the mass in relation to the great vessels and heart may have been the cause of the hypotension. The patient returned for a second stent placement, and computed tomography of the chest confirmed a cranial mediastinal mass, most suspected to be thymoma based on the results of cytology. The patient was kept in sternal recumbency, but when re-positioning to left lateral recumbency, there was a dramatic blood pressure drop that corrected with a return to sternal positioning.

**Conclusions:**

To our knowledge, orthostatic hypotension has not been described in relation to thymoma in dogs. Thymomas are rare; however, they may be associated with disease of autonomic dysfunction, such as myasthenia gravis, that may lead to orthostatic hypotension. This has been described within the human literature, and we hypothesize it was present in the currently described case. Concurrently, thymomas may grow to a substantial size and cause direct compression of the intrathoracic vasculature. As such, it should be on the differential list for poorly-responsive hypotension following a shift in body positioning under anesthesia.

## Background

Postural hypotension, also known as orthostatic hypotension, is described as autonomic dysfunction whereby the cardiovascular system’s adaptive mechanisms fail to respond to a reduction in venous return that is, in humans, associated with moving from the lying to upright position [8]. Prevalence in the human population is heavily age-dependent, occurring in over 30% of individuals greater than 70 years of age. Feelings of dizziness, light-headedness, and faintness are reported, and it is the second leading cause of syncope. Conversely, veterinary medicine has a paucity of information regarding symptoms or prevalence of orthostatic hypotension in canines. Hellyer et al. [5], looked at monitoring nerve conduction in 8 dogs, finding that there was a relationship between stellate ganglionic (sympathetic) and vagal nerve activity cessation with a blood pressure decrease upon moving from lying to sitting-up position. However, no dogs exhibited any signs of syncope or collapse and only 39% of the nerve conduction termination scenarios associated with blood pressure changes were related to movement and a change in position. Although mild cases in humans may respond to nonpharmacologic management, selective Beta2-blockers and alpha-adrenergic receptor agonists have been suggested and studied for more severe cases or to abate the blood pressure changes seen.

[6], studied 11 dogs with naturally occurring dysautonomia with presence or absence of postural hypotension and response to pharmacologic treatments. Dogs were restrained on an inclining table and blood pressure was compared between tilting a forelimb and hindlimb from horizontal to a 30–45-degree angle with thoracic limbs higher than pelvic limbs. Although only four dogs were studied, all showed evidence of postural hypotension that resolved when returned to a horizontal position, or when treated with phenylephrine. Interestingly, none of the affected dogs had a history of syncope or collapse. Dogs without dysautonomia were utilized as controls and compared to the study group; control dogs failed to experience significant blood pressure changes when inclined in the same manner.

Thymomas are a rare diagnosis in dogs, although it is typically diagnosed in older patients and large breeds, such as Labrador Retrievers, Golden Retrievers, and German Shepherds. Paraneoplastic syndromes, such as hypercalcemia or myasthenia gravis, have been associated with thymomas [4, 9, 11]. Postural hypotension is not commonly reported with thymomas, directly, but it has been reported with myasthenia gravis in humans as this is a disease of autonomic dysfunction [7]. Additionally, although rare, human case reports of heart failure and cardiovascular collapse secondary to large thymomas exist [3, 12].

## Case presentation

An eleven-year-old male, castrated, 47.2 kg Labrador Retriever presented to the Virginia-Maryland College of Veterinary Medicine Veterinary Teaching Hospital (VTH) in November 2018 for placement of a urethral stent. The patient had been diagnosed with transitional cell carcinoma via traumatic catheterization in January 2018, for which he had been seen monthly at the primary referral veterinary center to receive routine ultrasound examinations and chemotherapy (mitoxantrone 6.2 mg intravenously monthly) through October 2018. Over time the patient developed stranguria and there was evidence of thickening of the prostatic urethra in addition to a tumor present in the trigone. At presentation to the VTH, the patient was receiving piroxicam 17 mg q24h PO, milk thistle 175 mg q24h PO, S-adenosyl-methionine 400 mg q24h PO, omega fatty acid supplement 1600 mg q24h PO, and a vitamin B12 supplement.

From the time of initial diagnosis, routine monthly CBCs had been performed with no major abnormalities detected. Biochemical screening had been performed intermittently, with serial monitoring of BUN and Creatinine during chemotherapy treatment. At presentation for placement of the stent, the patient’s most recent bloodwork showed a slightly elevated BUN at 30 mg/dL (Reference Interval (RI) 7–27), elevated ALT at 159 U/L (RI 10–125), elevated ALKP at 981 U/L (RI 23–212), a slight neutrophilia at 13,110/μL (RI 2950-11,640), urine specific gravity 1.027, and a moderate amount of both blood and protein on free-catch urinalysis. Remaining values were unremarkable and within normal reference ranges. Physical examination revealed obesity (body condition score 8/9), mild to moderate hindlimb muscle atrophy, moderate dental tartar, several epidermal collarettes across the ventral abdomen, and a prominent urethra on rectal examination; all other findings were unremarkable. Ultrasound of the urogenital tract revealed thickening of the proximal urethral wall extending cranially into the neck of the bladder, punctate hyperechoic foci throughout the prostate, and a dilated left kidney pelvis and associated ureter.

Based on these findings, it was elected to perform cystoscopy with placement of a ureteral and/or urethral stent to relieve the stranguria. The patient was fasted overnight before anesthesia. Maropitant 1 mg/kg subcutaneously was administered prior to butorphanol 0.2 mg/kg intramuscularly and an intravenous catheter was placed to facilitate anesthetic induction. Induction consisted of intravenous fentanyl 5μg/kg followed by alfaxalone 1 mg/kg to effect. Following endotracheal intubation, the patient was maintained on sevoflurane in 100% oxygen with a fentanyl continuous rate infusion (CRI) at 10μg/kg/hr. and lactated ringer’s solution (LRS) at 5 mL/kg/hr. Anesthetic monitoring included capnography, pulse oximetry, esophageal temperature, electrocardiography (ECG), fractional expired sevoflurane (FESevo), fractional inspired oxygen percentage (FiO2), and direct arterial blood pressure.

Following placement of monitoring equipment, the patient was repositioned from sternal to dorsal recumbency in preparation for cystoscopy. At this time, mean arterial pressure (MAP) declined from 61 mmHg to 40 mmHg while the heart rate began increasing from 128 beats per minute (bpm). Sevoflurane was reduced while a fluid bolus of 10 mL/kg was initiated and a dopamine CRI started at 5μg/kg/min. The blood pressure did not improve, with the MAP around 45 mmHg, and the heart rate continued to climb to 180 bpm with the appearance of intermittent ventricular premature complexes. Lidocaine 2 mg/kg was bolused intravenously and a CRI at 2 mg/kg/hr. started while dopamine was increased to 10μg/kg/min. Sevoflurane administration was discontinued and the breathing system flushed with fresh oxygen. Blood pressure began to increase to a MAP of 70 mmHg with the heart rate lowering to 100 bpm and the patient began to move slightly, so fentanyl was increased to 15μg/kg/hr. while sevoflurane was restarted and maintained below FESevo 1.3%. As the patient returned to a surgical plane of anesthesia, the blood pressure decreased again, although less dramatically to a MAP of 65 mmHg. Dopamine was increased to 12μg/kg/min and a norepinephrine CRI at 0.1μg/kg/min was started. This ultimately provided a balance that maintained blood pressure while keeping the patient adequately anesthetized for cystoscopy and allowed the dopamine to be reduced to 5μg/kg/min. The patient was moved from dorsal to left lateral recumbency and hypotension returned, but it responded more quickly to increases in dopamine and norepinephrine than previously. Once stent placement was completed, the patient was put into a sternal position in the thorax with pelvic limbs laterally, at this point the blood pressure began to rise quickly to a MAP of nearly 90 mmHg, and both dopamine and norepinephrine were discontinued.

Two days after uneventful recovery and discharge, the patient presented to the primary veterinary referral center with epistaxis and an incidental cranial mediastinal mass was diagnosed on thoracic radiographs. The client did not wish to pursue further workup and Yunnan Baiyao was prescribed for the epistaxis. However, the stranguria that resolved with the stent placement returned over the following 2 months and it was elected to re-evaluate the first stent and potentially place a second stent. No further epistaxis episodes occurred.

The presence of a mass in the chest, confirmed via radiographs following discharge from the first visit, and the decline and then improvement in hemodynamics correlating to changing body position during the first anesthetic episode, made us believe that positioning was going to play a key role in management of blood pressure in the second anesthetic episode. The patient’s condition was largely unchanged from the first physical examination, and bloodwork was similar. Coagulation parameters were normal. Cytologic evaluation of an aspirate of the mass, obtained via thoracic ultrasound, was most suggestive of thymoma as reported by a board-certified veterinary clinical pathologist, who also recommended biopsy with histopathology for confirmation. Although this was not pursued by the client, computed tomography (CT) imaging was performed to rule out presence of a nasal mass and to better clarify size and exact location of the thoracic mass with respect to major vessels and the heart.

**Fig. 1:**
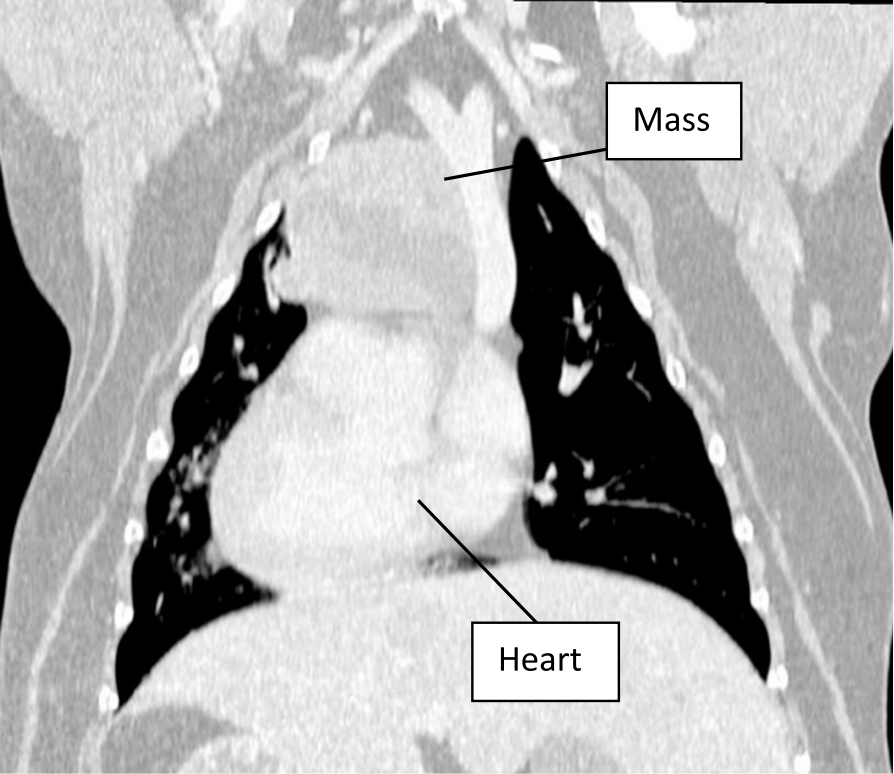
Dorso-ventral Computed Tomography Image. Note the mass just cranial to and in contact with the heart has a fluid-attenuating central portion and is causing deviation of the cranial vena cava

**Fig. 2:**
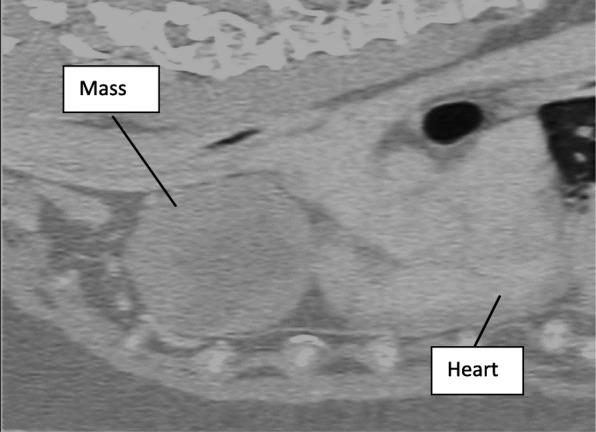
Sagittal Computed Tomography Image. Mass cranial to and in contact with the heart

The patient was premedicated with morphine 0.5 mg/kg and midazolam 0.2 mg/kg intramuscularly 1 h after receiving maropitant 1 mg/kg subcutaneously. Following sedation and catheter placement, induction was performed with a mixture of propofol 3 mg/kg and ketamine 3 mg/kg to effect. Propofol and Lidocaine CRIs were used for maintenance in place of sevoflurane, but the same monitoring as prior was utilized, including direct arterial blood pressure. Sternal recumbency was maintained throughout sedation, induction, and CT imaging. Due to the fast onset and short-acting vasoconstrictive effects of phenylephrine, it was administered in 1μg/kg boluses intravenously as needed for mild hypotension (MAP of 59-62 mmHg) during the early part of the anesthetic event, while the patient remained sternal. During this period, the blood pressure was responsive to phenylephrine and increased to a MAP of 65-75 mmHg shortly following boluses. The dramatic and poorly-responsive hypotension of the first anesthetic episode was not observed and the heart rate ranged from 80 to 110 bpm. CT imaging of the nasal cavity was unremarkable but it revealed a left cranial mediastinal, lobular, 7.5 cm (width) × 5.8 cm (height) × 6.8 cm (length) soft-tissue attenuating mass with an irregular fluid-attenuating central portion. It was noted to be causing rightward deviation of the cranial vena cava without direct vascular compression in the sternal position. For comparison, the heart measured 9.4 cm × 5.9 cm × 10.5 cm. Figures 1 and 2 illustrate a dorso-ventral and sagittal view of the mass as found on CT imaging.

Following CT imaging, the primary clinician requested left lateral recumbency for abdominal fluoroscopy and cystoscopy. As soon as the patient was re-positioned the mean arterial pressure dropped to 42 mmHg within 60 s. A dose of phenylephrine at 1μg/kg given intravenously failed to improve MAP in this instance. The patient was returned to sternal recumbency and MAP steadily increased back to its value prior to the change to lateral recumbency (MAP 63 mmHg). Ultimately the patient was maintained with the thorax sternal and the abdomen in oblique left lateral for the remainder of the procedure with no recurrent dramatic blood pressure changes.

## Discussion and conclusions

Although hypotension is commonly encountered under anesthesia in veterinary medicine, treatments aimed at increasing intravascular volume or modifying vascular tone often increase blood pressure. In the patient of the current report, these standard therapies did not consistently improve blood pressure as expected when the patient was in certain body positions. As such, we suspect that the intracranial mass was affecting the ability of the patient to respond via compression of intrathoracic vasculature and potential paraneoplastic myasthenia gravis, effectively causing blood pressure changes that related to body position.

In the case of the present report, the patient did not exhibit any signs of collapse or syncope outwardly, nor was there overt weakness of the gait, which if present may have been related to underlying disease such as myasthenia gravis. Per the client the patient had slowed down over time, but it had been attributed to age and obesity. Osteoarthritis is common in overweight and older dogs, although it was not evident in any series of radiographs for this patient; however, specific distal limb views were not taken. Similarly, megaesophagus was not found on imaging, nor was there a history of dysphagia. As the client did not wish to pursue further workup, acetylcholine receptor antibody testing was not performed, nor was mass removal and histopathology for definitive diagnosis of thymoma. The reported incidence of confirmed myasthenia gravis in dogs with thymoma varies from 0 to 46% [1, 2, 9, 13]. In humans, up to 50% of cortical thymoma patients develop myasthenia gravis [10], and so the potential contribution of it in this patient’s hypotension cannot be excluded, either.

A sudden and marked drop in blood pressure was seen with each time of changing position under anesthesia, but neither surgery involved significant elevation of the forelimbs in respect to the hindlimbs, which would mimic moving from a lying to standing position. Rather, each surgery involved rotating the patient from a sternal to lateral or dorsal recumbency only, but the head was kept elevated with respect to the stomach during the transition to reduce potential for regurgitation. Although this was a short period, it may have been enough for a change in venous return. Common blood pressure corrective measures did not seem to treat the hypotension as expected in the first anesthetic episode, and although phenylephrine prior to changes in body positioning in the second episode had controlled hypotension, it failed to control it after the change in recumbency. If entirely due to autonomic dysfunction and inappropriate constriction of blood vessels, the hypotension would have been expected to be treated by phenylephrine. However, as this was not the case, there is concern for a second component, such as direct vascular compression by the mass. This was not directly seen on imaging in a sternal position, but the deviation of the cranial vena cava that was demonstrated and sudden lack of response to phenylephrine may suggest it.

Multiple causes of orthostatic hypotension have been identified in human medicine, including but not limited to cardiovascular, endocrine, and autoimmune diseases. In the absence of an obvious cause or lack of testing, cases may be listed as idiopathic [8]. Routine bloodwork and historical patient information failed to provide an obvious cause for the hypotension in this patient, aside from a potential effect secondary to the suspected thymoma. As such, computed tomography imaging of the thorax was requested to determine if there was direct compression of the great vessels, which was not observed on CT imaging in sternal recumbency. It is most suspected that there was a pronounced blood pressure decrease via the combination of decreased venous return from mass compression of the vena cava when switching positions and potential paraneoplastic autonomic dysfunction; however, lack of additional testing makes a definitive answer difficult for this patient.

## Data Availability

Not applicable, Case Report.

## Abbreviations

VTH: Veterinary Teaching Hospital
RI: Reference Interval
CRI: Continuous Rate Infusion
LRS: Lactated Ringer’s Solution
ECG: Electrocardiography
FESevo: Fractional Percentage Expired Sevoflurane
CT: Computed Tomography
MAP: Mean Arterial Pressure
